# Medical Student-Perceived Barriers, Facilitators, and Considerations Regarding Clinical Empathy Training: A Qualitative Study

**DOI:** 10.1177/23821205261484413

**Published:** 2026-09-06

**Authors:** Shira Gertsman, Anushka Nair, Jason Wang, Connie Li, Sarah Klapman, Johanna Shapiro, Connie Williams

**Affiliations:** 1 Michael G. DeGroote School of Medicine, 3710 McMaster University, Hamilton, ON, Canada; 2 Department of Family Medicine, School of Medicine, 8788 University of California Irvine, Irvine, CA, USA; 3 Department of Pediatrics, Faculty of Health Sciences, 3710 McMaster University, Hamilton, ON, Canada; 4 The Dalla Lana School of Public Health, 274071 University of Toronto, Toronto, ON, Canada

**Keywords:** empathy, medical education, medical students, clerkship, hidden curriculum

## Abstract

**Introduction:**

Clinical empathy involves understanding a patient’s illness experience and acting on it to provide collaborative care. Despite evidence that clinical empathy leads to improved patient outcomes and physician wellbeing, clinical empathy declines in medical students during training. The primary objective of this study was to explore medical students’ perspectives on how elements of medical education influence their clinical empathy.

**Methods:**

We conducted a qualitative focus group study using adapted constructivist grounded theory methodology. Students from Ontario medical schools took part in semi-structured virtual focus groups between April 2022 and March 2023. Transcripts were coded using constant comparison and adapted into visual networks to conceptualize students’ experiences with clinical empathy training. Feedback on current curricular elements was analyzed descriptively.

**Results:**

Twenty students participated in five focus groups. The resultant conceptual model illustrated that students learn empathy mostly from structured teaching in preclerkship and the ‘hidden curriculum’ in clerkship. How these experiences affect empathy depends on personal factors (inherent empathy, wellness, cognitive load), preceptor factors (modelling, specialty, behaviour toward learners, burnout) and school factors (curriculum, assessment). Students identified targets for improvement, including integrating humanistic and medical teaching, involving patients in education, promoting interdisciplinary experiences, and teaching psychologically-safe empathy practices.

**Conclusion:**

Although educational interventions targeting empathy can have positive effects, they can prompt students to deprioritize empathy if implemented incorrectly. A focus on student well-being, supportive learning environments, and empathetic preceptor behaviour is necessary for students to yield optimal benefit from these interventions.

## Introduction

Clinical empathy, as defined by Mercer and Reynolds, is a physician’s ability to “(a) understand the patient’s situation, perspective, and feelings (and their attached meanings); (b) to communicate that understanding and check its accuracy; and (c) to act on that understanding with the patient in a helpful (therapeutic) way”.^1^ Clinical empathy is cognitive and behavioural in nature and does not require affective empathy (i.e., feeling what the patient is feeling).

Patients who are treated with clinical empathy are more likely to trust their physician and adhere to their treatment plan, cope better with their illness, and have improved physical and mental health outcomes.^2-5^ Clinical empathy also bolsters physician wellness, reduces litigation, and improves healthcare resource stewardship.^5-10^ In light of the established benefits of clinical empathy,^2-10^ international medical education bodies have integrated empathy into their learning objectives and frameworks for medical professionalism.^11-13^ Yet, medical students’ clinical empathy has been shown to decline during clinical training.^14-16^ Although medical students’ knowledge of and ability to enact medical interventions increases over time, the understanding and communication elements of clinical empathy, which do not necessarily increase with biomedical skill acquisition, are crucial to ensure that medical treatment is conducted in line with patients’ wishes, goals, and accessibility needs.^5^

Educational interventions have been proposed to enhance medical student empathy, including didactics, small-group discussions, reflective exercises, simulations, creative arts, and more.^16-18^ Curricula with direct patient involvement, such as patients sharing their experiences, longitudinal student-patient pairs, and visits to homes of patients with disabilities, have shown positive effects.^16,19^ Yet, a preponderance of learning occurs implicitly through experiences and observations. This so-called ‘hidden curriculum’ imparts values, norms, skills, and knowledge to medical students, which shape their clinical attitudes and behaviours, including clinical empathy.^20^ Furthermore, numerous factors are postulated to inhibit clinical empathy, including student distress, inadequate role models, fragmented clinical relationships, and psychological defense against vulnerability to illness.^14,15,21^

Effectively integrating clinical empathy-promoting interventions into medical education requires an understanding of the relationships between the formal curriculum, informal ‘hidden’ curriculum, and barriers to clinical empathy. Student perspectives on how these factors affect their learning of clinical empathy are lacking in existing literature and essential to guide innovation in this area. Therefore, we asked the following question: “What can be learned from Canadian medical students about how formal and informal elements of medical education influence clinical empathy?”

The primary objective of this study was to explore medical students’ perspectives on how formal and informal elements of medical education influence their clinical empathy. The secondary objectives were to summarize student feedback regarding existing clinical empathy-promoting curricular elements and to gather student input on how clinical empathy training can be improved.

## Methods

This study was part of the Empathy in Medical Professionals: Augmenting Curriculum and Training (EMPACT) Project,^5^ a student-led initiative that sought to improve physician-patient relationships by informing innovation in medical education. The reporting of this study conforms to the Standards for Reporting Qualitative Research [Supplementary File 1].^22^

### Researchers, Reflexivity, & Rigour

The research team comprised five medical students and two supervising professors who had extensive experience with design, performance, and supervision of qualitative research. For details on how awareness of how researcher experience, positionality, and reflexivity influenced coding categories and data interpretations, please see Supplementary File 2, Appendix A.

### Ethical Issues

All research activities were approved by the Hamilton Integrated Research Ethics Board (#12912). Given the sensitive nature of the interview content, participants were instructed to avoid identifying individuals when sharing stories. Participants were aware that the focus group might contain their classmates and were explicitly allowed to keep their video off, change their name, and/or use the chat function for anonymity. They could also skip questions or leave the group at any time. A group debrief and an option for individual feedback/discussion (anonymous or identified) were both offered after each group, and links to student support services at the applicable schools were provided.

### Study Design

To address our primary objective, we employed an interpretive qualitative methodology drawing from Charmaz’s constructivist grounded theory (GT).^23^ Grounded theory is an inductive methodology whereby theories and concepts are derived directly from the data, without a predetermined hypothesis, allowing for generation of new ideas regarding poorly studied topics.^23^ However, traditional GT is designed for advanced theorizing, and modifications may be required for smaller scale, applied research.^24^ Given our aim to generate practical recommendations for medical education, our methods were based on fundamental processes and philosophies of GT with modifications to optimize the practicality of our results.

Many fundamental elements of GT methodology were used in study design. Most importantly, our results were grounded in participants’ first-hand experiences. To achieve this, we avoided pre-determined hypotheses or rigid conceptual frameworks, and used Mercer and Reynold’s definition of clinical empathy as a sensitizing concept.^1^ Iterative and interpretive analysis strategies using constant comparison, described below, were also true to constructivist GT. Sampling method was purposive, specifically selecting students from different schools at different levels of training to maximize variation. In line with Charmaz’s GT approach, through our constructivist research paradigm, we viewed knowledge as constructed by the interplay between researchers (medical students and medical educators) and research participants (medical students).^23^

To address our primary research question, we modified GT methods by aiming to construct a “conceptual description” of students’ perspectives, rather than an all-encompassing “theory”.^24^ Instead of relying on theoretical saturation as a recruitment endpoint, which may not be achieved through a smaller scale project, we employed “information power” for sample size estimation (described below).

For our secondary objective, which was purely descriptive, we used manifest content analysis to summarize current curricular interventions and student suggestions for improving clinical empathy training.^25^

### Sample Size & Sampling Methods

We recruited participants by advertising the study on student Facebook pages and newsletters for each medical school in Ontario, Canada. The Northern Ontario School of Medicine was eventually excluded due to a lack of participant volunteers. Volunteers completed a screening survey and were eligible if they were at least 18 years old, spoke English, were matriculated at an accredited medical school in Ontario, and completed at least one full year of medical school.

The recruitment endpoint was estimated based on information power, a descriptive measure incorporating attributes of study objectives, sample, dialogue, and analysis.^26^ To ensure all participating schools were represented in the analysis, we required at least one focus group per school. Students from the same school were grouped together to optimize discussion of shared experiences. To diversify participant experiences, students were purposively sampled to include at least one final-year clerk and one student in preclerkship/early clerkship in each group. Based on focus group literature,^27^ we predicted that one group per school (5 groups, totalling 15-25 participants) would provide sufficient information power to meet our exploratory aims, presuming adequate quality of dialogue.

### Data Collection

Focus groups were held between April 2022 and March 2023. The focus group guide (Supplementary File 2, Appendix B) was piloted on four first year medical students who provided feedback on question relevance and clarity, which was used to improve the moderator guide. Each participant completed a questionnaire collecting demographic information (Supplementary File 2, Appendix C). The semi-structured groups lasted two hours on Zoom© and were co-moderated by two members of the study team: one moderator asked prompting questions from the guide; the other asked clarifying questions, made live memos to enrich interpretation, and ensured consistency across groups. Mercer and Reynolds’ definition of clinical empathy was explained to participants, provided in writing, and revisited frequently.^1^ Participants were given freedom to determine which curricular elements they felt were congruent with this concept. Focus groups were recorded and transcribed verbatim.

### Data Analysis

Participant demographics were summarized using descriptive statistics. Qualitative analysis was performed manually using ATLAS.ti 23.2.1 for Mac.^28^ Concurrent with data collection, two study team members independently performed initial line-by-line coding of each transcript using the constant comparative method,^23^ then conferred to reflexively discuss codes and refine categories together. Live memos taken during focus groups were reviewed from an interpretive lens for any meanings not already captured within existing codes, which were added as new codes when identified. As predicted, sufficient information power for the exploratory and conceptual descriptive aims was reached after coding one focus group per participating school (five groups total).^26^ No new themes were generated from line-by-line coding of the final two transcripts, which were each reviewed by two independent reviewers who compared their individual codes with the existing code list and, after initial coding, with one another. We performed member-checking by sending codes and subcodes to participants for their feedback (Supplementary File 2, Appendix D), which was subsequently incorporated into analysis.

We performed inductive focused coding by reviewing all quotations under each code and organizing the codes into larger categories and smaller sub-codes, using constant comparison throughout.^23^ We then used a constructivist approach to axial coding,^23^ whereby codes and categories were visually and directionally linked using ATLAS.ti Networks.^28^ The visual networks were used to develop a conceptual model of the students’ perspectives on clinical empathy teaching in medical school.

To address our secondary objectives, we performed manifest content analysis by compiling quotations describing elements of existing medical school curricula and relevant feedback and suggestions. These elements were grouped into categories and presented descriptively.

## Results

### Participants

Because recruitment occurred through open advertisement, the number of students who viewed the invitation and chose not to respond is unknown. Sixty-two individuals submitted the complete screening form, which required meeting full eligibility criteria. Twenty-four completed the consent procedures and, in the end, 20 students participated across five focus groups. No participants who were willing and available to attend were excluded by the study team. Each group was scheduled with three to five participants from the same school, although one contained only two participants due to cancellations. Participants received an incentive of $30 CAD for attending the focus group. Participant demographics are summarized in Table 1.

**Table 1.** Participant Characteristics According to Responses on the Demographics Survey

Characteristics	Number of Participants, *n* (%)
Gender identity	
Woman	17 (85%)
Man	2 (10%)
Prefer not to say	1 (5%)
Age (years)	
20-24	8 (40%)
25-29	10 (50%)
30-34	2 (10%)
Medical School	
McMaster University	4 (20%)
Queen’s University	5 (25%)
University of Ottawa	4 (20%)
University of Toronto	2 (10%)
Western University	5 (25%)
Level of training	
Preclerkship	8 (40%)
Early clerkship (not yet in final year of medical school)	5 (25%)
Late clerkship (in final year of medical school)	7 (35%)
Identification as visible minority/minorities or other marginalized group(s)	
Yes	11 (55%)^a^
No	7 (35%)
Prefer not to say	2 (10%)

^a^Participant self-descriptors included the following: POC, Black, Hijabi, Asian, Chinese, immigrant, first-generation physician, single income family, and Jewish.

Only one participant provided feedback in member-checking. The feedback was positive with a suggestion of reorganizing one subcode into higher level codes that they felt were more reflective of the subcode’s meaning, which was accordingly incorporated into our code organization. It is unknown if those who did not respond failed to review the codes or simply did not have any specific feedback.

### Student Learning of Clinical Empathy

**Figure 1.**
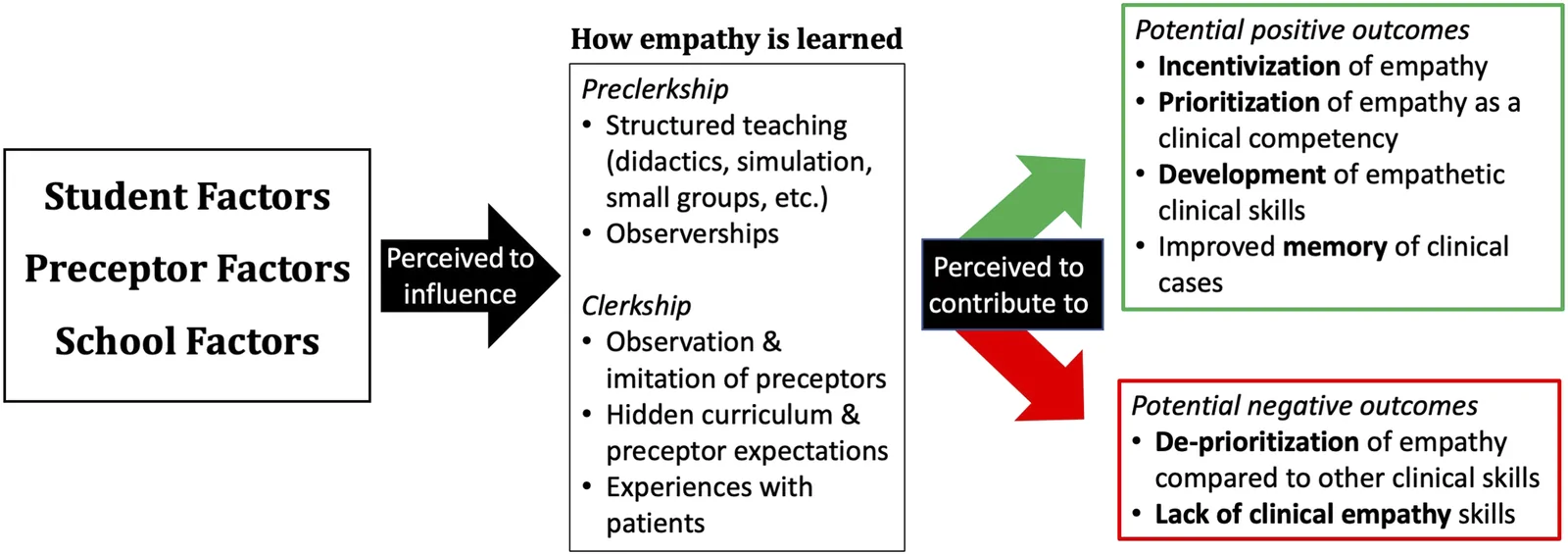
Medical students’ perceptions of how student, preceptor, and school factors may influence the effects of various medical school experiences on their clinical empathy. Learning opportunities were perceived as having the potential to enhance or diminish clinical empathy in students depending on the student, preceptor, and school-related factors

Students identified three main categories of factors that they perceived as affecting their ability to learn clinical empathy: student factors, preceptor factors, and school factors (Table 2). In concert, these factors were thought to determine the effects of students’ formal and informal learning experiences relating to clinical empathy, which consisted mostly of structured teaching in preclerkship and preceptor modeling and patient experiences in clerkship (Figure 1). Learning opportunities were perceived as having the potential to enhance or diminish clinical empathy in students depending on the student, preceptor, and school-related factors.

**Table 2.** Factors That Students Believed Influence Their Clinical Empathy in Medical School, as Shown in Figure 1. Arrows Indicate a Positive (↑) or Negative (↓) Association With Effective Learning of Clinical Empathy

Factors Influencing Clinical Empathy Learning	Examples & Directions of Influence
Student Factors	• Inherent empathy (↑) • Personal wellness (↑) • Fatigue, cognitive load, burnout (↓)
Preceptor Factors	• Modelling clinical empathy (↑) • Taking time to explicitly teach communication skills (↑) • Demonstrations of empathy toward learners and colleagues (↑) • Sharing personal vulnerability with learners (↑) • Safety of the learning environment (↑) • Medical specialty (↑/↓) • Preceptor expectations of empathy in learners (↑/↓) • Harsh criticism of learners (↓) • Preceptor burnout (↓) • Time constraints and high patient loads (↓)
School Factors	• Prioritization in formal curriculum (↑) • Sincerity and “practicing what they preach” (↑) • Feedback, assessment & evaluation (↑/↓) • Clinical format of learning in clerkship (↑/↓) • “Checking boxes” for accreditation (↓) • Multiple campuses & many clerkship sites (↓) • Empathy training siloed from pathophysiology/medical teaching (↓)

#### Student Factors Affecting Learning of Clinical Empathy

Student factors were strongly emphasized (Table 2). Participants postulated that higher inherent empathy may positively correlate with receptivity to curricular interventions:

*“I feel like the people who excel in the classroom are also people who are just naturally empathetic people, they just care about other people, versus people who are just like, ‘Oh, it’s something we need to get done.’”* (School D Participant)

Although high baseline empathy was primarily viewed as a facilitator of learning clinical empathy, some students believed that it may precipitate cognitive distancing from patients in clinical situations out of concern for students’ emotional distress.

*“As a really empathetic person in general, I'm really deeply affected by others’ situations, […] and I found that actually clerkship has been one of the experiences in my life where I’ve experienced a little bit less of it personally and I think it's been more of a defense mechanism.”*(School B Participant)

In addition to baseline empathy, participants emphasized that they perceived students’ responsiveness to clinical empathy teaching as strongly influenced by modifiable factors including cognitive load, fatigue, and burnout, which are dependent on other elements of the curriculum and medical school supports.

#### Preceptor Factors Affecting Learning of Clinical Empathy

Clinical preceptors were described to significantly affect students’ prioritization of clinical empathy through the preceptors’ behaviour toward students, patients, and allied health professionals (Table 2). This was experienced as most pronounced during clerkship.

*“[My preceptor] really ruined my confidence, my self esteem […] and that really did impact how I communicated with my patients.”* (School C Participant)

Preceptor modelling of empathy toward patients was perceived by students as a ‘hidden curriculum’ of lessons, values, and expectations. Perceived helpful behaviours included debriefing, setting appropriate professional boundaries, and portraying an empathetic example for the entire care team, while perceived harmful behaviours included talking negatively about patients in private.

Students acknowledged that preceptors’ clinical empathy may be influenced by similar personal factors as their own, including their inherent empathy, desensitization, and burnout, as well as external factors, including time constraints, pay structure, and medical specialty. Psychiatry, palliative care, and oncology were considered by participants to be specialties where they observed more clinical empathy, which students attributed to the ability to spend more time with patients and have longitudinal follow-up; emergency medicine, general surgery, orthopedics, radiology and pathology were experienced by students as exhibiting less clinical empathy, which students attributed to higher acuity and limited physician-patient interaction.

*“I think that the workplace culture also impacts it, because even across different specialties and models, the specific culture, what’s accepted, and the behaviors that get rewarded, I think that plays into it as well.*” (School C Participant)

Psychiatry was identified as a specialty in which debriefing was felt to be prioritized and preceptors offered language, such as “countertransference,” to help students understand their feelings toward patients; students felt empowered by this to normalize negative feelings and learn how to process them to prevent negative behaviours toward patients:

*“[My preceptor] was like, ‘You felt that counter-transference too, right?’ There’s medical terminology and it tells me about that patient and about myself. And that was so validating, to be like, ‘I’m not a bad person for being irritated by this patient, […] it’s okay for me to feel that way but we still are going to provide the therapeutic care.’”* (School C Participant)

Students observed that certain patient attributes, such as agitation, anger, and anxiety, could make it challenging for some preceptors to display empathy.

#### School Factors Affecting Learning of Clinical Empathy

Factors related to medical school and curriculum structure were also perceived by students as affecting learning of clinical empathy (Table 2). Prioritization, integration, and sincerity of empathetic content in curricular instruction and assessment were experienced as having positive effects. However, the structure of clerkship rotations was seen to limit the ability to provide standardized empathy teaching beyond preclerkship. Furthermore, students reported struggling to fulfill conflicting roles as learners and service-providers, particularly in high-volume healthcare settings where productivity may be prioritized over education.

The discontinuous nature of clinical rotations, requiring medical students to constantly adjust to new clinical settings and form new relationships with staff who have variable expectations and priorities, was viewed by students as hindering the ability to learn humanistic skills by placing significant cognitive load on learners. Students also reported that the disorientation experienced when changing clinical specialties increased their reliance on modelling preceptor behaviours to function in the new environment.

### Teaching Clinical Empathy

Students highlighted numerous elements of their curricula that they believed influenced clinical empathy and provided feedback on these methods (Table 3).

**Table 3.** Existing Elements of Ontario Medical School Curricula That Students Believed Influence Their Clinical Empathy. Use of These Elements Varied Across Schools. Elements Are Listed in Alphabetical Order

Curricular Element	Examples	Student Feedback
Assessment	⁃ OSCE stations that assess empathetic communication, such as “breaking bad news” ⁃ Ratings of communication/empathy skills in clerkship	⁃ Reinforces concepts ⁃ Prioritized by students ⁃ Can feel artificial, such as saying “one-liners” to “get the mark” ⁃ Evaluation in clerkship is often generic and non-specific
Case-based learning	⁃ Using patient cases to learn about medical conditions	⁃ Perceived as helpful to some, while others did not find it motivating
Debriefing	⁃ Group debrief sessions formally incorporated as protected time during clerkship rotations ⁃ Mandatory annual sessions with Wellness Counsellors ⁃ Semi-structured small group reflections facilitated by a physician	⁃ Helps students feel supported ⁃ Viewed as schools “practicing what they preach” regarding empathy
Didactics	⁃ Lectures about patient perspectives and how to support patients in their health journeys ⁃ Didactic teaching with examples of how to phrase difficult news and navigate patients’ possible emotions ⁃ Talks by palliative care physicians ⁃ Slideshows with definitions and tips for practicing empathy and self-care	⁃ Helps to see modelling of how to conduct difficult conversations ⁃ Helps when physician-lecturers give personal examples of difficult patient scenarios they have navigated ⁃ Would like patient experiences to be integrated into didactic teaching about medical conditions (currently it is often separated) ⁃ Lectures about how to be empathetic feel like a “checkbox” without actionability ⁃ Students are often not engaged with this teaching as other parts of education viewed as higher stakes ⁃ Perceived as counterproductive when mandatory wellness lectures take away from personal time
Interdisciplinary education	⁃ Shadowing allied health on shifts ⁃ Interviewing allied health team members	⁃ Allied health often has better insight into patients’ lived experiences, especially when they perform home care, and are good teachers of this ⁃ “Mind-blowing” to learn how different allied health team members fit into patient care
Media	⁃ Online resources with first-person patient stories ⁃ Patient documentary viewings	⁃ Helps students understand healthcare system from patient perspective ⁃ Perceived as important to have online patient perspectives available when encountering diseases clinically, though students rarely referenced them in practice
Models of clinical empathy	⁃ FIFE (Feelings, Ideas, Functioning, Expectations)^a^ ⁃ SPIKES model for breaking bad news^b^ ⁃ Kleinman’s Explanatory Model for understanding patients’ perspectives^c^	⁃ Highlights the patient’s perspective during clinical skills teaching ⁃ May not feel practical to use in real life; however, observing skilled preceptors using models effectively in practice helps students translate the skill to the clinical environment
Patients sharing experiences	⁃ Patients speaking about their diagnoses and life with their medical conditions ⁃ Patients speaking about intersectional experiences (e.g., poverty, violence, houselessness) and identities (e.g., Indigenous, 2SLGBTQIA+) ⁃ Clinical skills sessions interviewing patients with lived experience	⁃ Seen as stimulating and engaging, evoking emotional responses in students ⁃ Allows students to better understand experiences of patients they later see on clinical rotations ⁃ Facilitates better memory of the medical conditions ⁃ Demonstrates what patients value in physicians ⁃ Usually siloed from medical teaching of disease, more effective when integrated ⁃ Not well-attended if optional ⁃ Can be awkward if patient interactions are virtual
Patient partnering	⁃ Being matched with a patient who you accompany to appointments (with physicians and allied health) longitudinally to learn about their healthcare journey	⁃ Increases student awareness of things they had not considered: e.g., waiting for 6 hours for a 10-minute specialist appointment, seeing how their illness impacts their daily life, observing interactions with allied health, etc.
Shadowing	⁃ Shadowing physicians in preclerkship	⁃ Helps to observe skilled preceptors navigating challenging conversations
Simulation	⁃ Standardized patients (SPs) who give feedback on communication skills	⁃ Helps to have feedback from actors ⁃ Helps to have setting where you can focus on communication skills without being evaluated on medical knowledge ⁃ Can feel artificial to express empathy toward SPs
Small group learning	⁃ Semi-structured small group discussions regarding humanistic aspects of medicine such as social determinants of health and patient perspectives	⁃ Encourages understanding of patients’ perspectives and social histories

^a^Weston WW, Brown JB, Stewart MA. Patient-centred interviewing part I: understanding patients’ experiences. Can Fam Physician. 1989 Jan; 35:147-51. https://pmc.ncbi.nlm.nih.gov/articles/PMC2280441/

^b^Baile WF, Buckman R, Lenzi R, Glober G, Beale EA, Kudelka AP. SPIKES-A six-step protocol for delivering bad news: application to the patient with cancer. Oncologist. 2000;5(4):302-11.https://doi.org/10.1634/theoncologist.5-4-302

^c^Kleinman, A. Culture, Illness, and Care: Clinical Lessons from Anthropologic and Cross-Cultural Research. Annals of internal medicine 1978;88(2);251–258. https://doi.org/10.7326/0003-4819-88-2-251.

In general, patient-led interventions such as patients sharing their experiences or patient-partnering were viewed favourably and characterized as evoking authentic emotions that organically engendered empathy formation. This natural empathetic response was perceived to improve memory of clinical information when presented together, such as a patient sharing their personal story at a lecture teaching about their illness:

*“We had a 10-year-old come in and talk to us about his congenital heart disease with his mom, and it was so emotional and amazing. […] I also remember a lot about his heart condition better because we spoke about it with him.”*(School B Participant )

Interdisciplinary education and debriefing were also viewed as effective means of clinical empathy enhancement. In contrast, students felt that clinical empathy is difficult to teach didactically. Although didactics and standardized patients can play an important role, their contrived nature was considered to be limiting.

*“With OSCEs and standardized patients I just find it really awkward to be empathetic because it sounds fake, because you have to just say it so that you get the point.”*(School A Participant)

Students offered suggestions for curricular innovation in this area (Table 4). They emphasized that didactic teaching in preclerkship can be used to build an awareness of barriers to clinical empathy, but they viewed modelling by preceptors as the most important teaching tool in the clinical setting. Students believed didactic teaching of patient experiences should be *integrated* into conventional medical teaching rather than separated, to optimize both the benefits to students’ clinical empathy and their consolidation of clinical material. Students also desired designated time to talk to patients, such as while the patients are waiting to see the clinician, to learn about their stories and experiences. Students expressed a need for more clinical experiences with interdisciplinary team members, as these individuals’ work often involves understanding patients’ lives outside the clinical setting. Furthermore, students reported missing instruction on methods of practicing empathy in a mentally and emotionally safe way, such as how to balance empathy, boundaries, and compartmentalization.

**Table 4.** Student Suggestions to Improve Clinical Empathy Training in Medical School

Category	Suggestions
Clinical experiences	• Include educational experiences with allied health & social service providers • Have skilled preceptors demonstrate clinical interviews and difficult discussions before students attempt with real patients • Designate time to spend simply talking to patients on less-busy rotations or in preclerkship shadowing experiences
Didactics	• Emphasize palliative and patient-centred options and goals of care, in addition to active/curative medical treatments • *Integrate* patient experiences into didactic “medical” teaching, preferably through patients’ own voices • Teach how to cope with own emotions as a practitioner • Teach how to practice clinical empathy in a mentally/emotionally safe way, exploring the balance between empathy, boundaries, and compartmentalization • Equip students to recognize non-empathetic behaviour in preceptors and respond in ways to avoid adopting that behaviour • Teach about barriers to clinical empathy to prepare students for observations in clinical settings
Preceptors	• Implement a method of reporting and remediating or removing preceptors that perpetuate toxic/inappropriate work environments • Remind preceptors to lay out clear expectations at start of rotations to reduce student stress and cognitive load
Simulation	• Include patients with intersectional identities in OSCE stations
Student wellness	• Build formal debriefing into rotations during protected time to allow students to share their experiences with their peers • Offer mental health days for student wellness • Provide support and empathy for students going through medical training to reduce their burnout and overwhelm
General advice	• *Consistently* prioritize and integrate patient perspectives to emphasize their importance rather than restricting them to specific sessions • Incorporate these suggestions into the learning schedule rather than adding mandatory sessions that take time away from self-care • Consider that empathy-focussed sessions may or may not be more effective when optional versus mandatory o When mandatory, students may feel resentment and disinterest o If optional, those who would benefit the most may not attend

Importantly, students’ curricular recommendations were still perceived as influenced by the student/preceptor/school factors shown in Figure 1. For example, while patient experiences were highly valued as builders of empathy, challenging patient experiences could lead to deprioritization of empathy if complicated by student burnout and lack of preceptor support. Several participants also expressed uncertainty regarding to what degree empathy can be learned versus being inherent to the individual, and one participant expressed the opinion that it is not a teachable skill.

## Discussion

Our student-derived model is supported by previous models of medical student empathy^14^ and identifies important new hypotheses, specifically regarding the interaction of barriers and facilitators of clinical empathy learning. While students acknowledged the contribution of the formal curriculum to clinical empathy training, the learning outcomes were perceived to depend on student and preceptor factors. Correspondingly, Winseman and colleagues found that “mentoring and clinical experiences that promote professional growth” affected clinical empathy more than explicit educational experiences.^29^

This does not mean that school factors do not play a role – interpretive structural modeling research demonstrates that the physician behaviours and interpersonal relationships within the hidden curriculum are themselves dependent on organizational factors. Thus, in accordance with systems theory,^30^ addressing higher-level institutional issues may have significant ripple-effects on preceptor behaviours and, subsequently, on student learning.^20^ Student factors that were perceived to influence receptivity to clinical empathy training, such as distress and burnout, were also reported to be directly affected by the structure and content of medical training. This suggests that schools must target improvement of learning conditions as an indirect method of sustaining student clinical empathy, and that the additional workload created by adding extra empathy-promoting sessions without modifying existing curricular requirements may be counter-productive.

System factors may also explain students’ reflections that they observed more clinical empathy in certain specialties. International studies have suggested that medical students interested in “people-oriented specialties” have higher empathy scores than those interested in “technology-oriented specialties”,^14,31,32^ and that this persists in licensed physicians, with particularly high empathy in psychiatrists.^33^ The strong role of preceptors in empathy development may perpetuate this trend: students are more drawn to specialties when they have similar traits to physicians and residents in that field, and model the behaviours of their mentors. However, this does not indicate that individuals who pursue certain fields are inevitably less empathetic; as some participants pointed out, differences observed are more likely related to cultural and institutional/logistical factors such as time with patients, physician burnout, acuity of patients’ illnesses, and ability to form longitudinal patient-physician relationships.^15^ These factors may also explain why students highlighted allied health professionals, who are often able to spend more time with patients longitudinally, as strong teachers of clinical empathy.

The notion that clinical empathy is reduced in both students and preceptors when they are experiencing psychological distress recapitulates the findings of previous studies^14,21^ and has a physiological basis in mirror neurons, which are impaired by stress, anxiety, and callousness.^14^ Interestingly, students’ speculations that higher clinical empathy may be associated with increased burnout opposes what has been demonstrated in the literature.^6-8,10^ Personal distress related to empathy generally relates to *affective* empathy (i.e., feeling another’s feelings)^34^ which has not been shown to have significant clinical benefit.^10,35^ Clinical empathy, however, comprises only *cognitive* (understanding patients’ feelings) and *behavioural* (communicating and acting on understanding) components of empathy. Understanding this distinction may reassure students concerned about the effects of clinical empathy on their emotional wellness. There is growing literature on the importance of emotion regulation and emotional intelligence in physicians,^36,37^ and our results suggest a need for instruction on how to practice clinical empathy in a psychologically safe manner.

A recurring hypothesis shared by participants was that the effectiveness of clinical empathy training may depend on students’ baseline empathy. Evidence supports this: higher baseline empathy scores are associated with greater pre–post gains following educational interventions.^18^ Some attributes of baseline empathy described by participants, namely emotional responses, seemed to reflect affective empathy. While affective empathy is not a required component of clinical empathy, higher baseline affective empathy is likely associated with higher cognitive/behavioural empathy, and the emotional response evoked by affective empathy may prompt a student to place more importance on developing clinical empathy skills. In didactic or simulation settings, explicit labeling of activities as “empathy training” may prompt disengagement among less empathy-oriented students. In contrast, learners believed that emotionally authentic experiences—such as hearing patient narratives or observing real clinical interactions—are more effective at fostering clinical empathy.

### Student-Informed Guidance for Educators

Participants provided recommendations on how they believe teachers and institutions can foster clinical empathy in medical students (Tables 3 and 4), and our findings raise several hypotheses for curricular design.

Students emphasized the importance of baseline empathy on their development of clinical empathy, indicating that it may be important for medical schools to consider creative and effective ways to fairly assess clinical empathy in prospective students; currently, many schools internationally use measures such as the Computer-based Assessment for Sampling Personal Characteristics (CASPer),^38^ Multiple Mini Interview,^39^ or other situational judgement tests as a proxy for these traits. However, such standardized measures have limitations and may bias against minority applicants, highlighting the need for reevaluation of these methods.^40^

Students suggested that their clinical empathy can be potentiated or hindered by preceptors. Knowing that preceptors’ ability to model empathy may be influenced by the same personal and institutional factors as students’, appropriate support, training and evaluation of clinical preceptors may facilitate experiential learning of empathy. Changes at institutional and cultural levels are necessary both to enhance students’ empathy capacity directly and indirectly through enhancing that of preceptors. Observation of clinical empathy in preceptors could be enhanced by interpretive exercises where students may reflectively identify what components of the preceptors’ behaviour were effective. Other helpful interventions may include Balint groups or regular debriefing sessions among preceptors and students,^41^ which can help students cope with distress and process challenging clinical experiences without attaching negative meaning to patients.^15^

Students perceived that their wellness and cognitive load influence their receptivity to learning and practising clinical empathy. Thus, it may be important for schools to prioritize support for students to minimize burnout and distress; adding didactic sessions on clinical empathy may meet accreditation requirements but, by taking away from students’ time for rest and self-care, it may counterintuitively deprioritize empathy development.

Patient involvement in teaching and curricular design was strongly recommended by medical students and can be integrated into existing teaching; for example, a patient with a medical condition can speak live at the lecture on that condition, or authentic patient narrative videos can be included.^42^ If feasible, longitudinal patient-partnering and home visits seem to be particularly effective due to their immersive qualities. Furthermore, allied health professionals such as occupational therapists should be involved in curricular development and teaching in clinical/non-clinical contexts, as they often get to know patients more holistically outside of conventional medical settings.

### Limitations and Strengths

While the sample size of this study is relatively small, it is appropriate for the exploratory objectives and qualitative methodology. Our aims are intended as a starting-point for future research, innovation, and implementation, and do not imply comprehensive representation of medical students. Although localized to Ontario, the five included medical schools are situated in locations with distinct populations and environments resulting in diverse student experiences.

The results of this study are shaped by participant self-selection bias, as student characteristics and attitudes fundamentally shape their experiences of empathy and are the foundation of our data. It is likely that the students who volunteered to participate in this study are interested in clinical empathy, making it difficult to identify educational strategies for students who place less importance on the topic. The absence of participation from the only rural institution in Ontario (NOSM) is notable as not only do we lack the perspectives of rural trainees but, also, it is unclear if the reason for their absence is a result of less interest in this topic or other individual or structural barriers to participation. The majority-female participant group in this study is likely a result of the self-selection bias, as there is evidence that women medical students may have higher empathy scores than men;^32,33^ importantly, this is more likely related to socialization of women students toward empathetic behaviours and people-oriented specialties rather than inherent gender differences. No gender diverse individuals participated, which may reflect the underrepresentation of this group in medical education.^43^ Cultural background may also significantly influence how empathy is prioritized, defined, and enacted; while over half of participants identified as members of minority groups, these nuances were not explored in detail.

Strengths of this study include its GT-based qualitative methodology, which permitted us to construct new ideas directly from participants’ stories while focusing on pragmatism of results. The use of medical student interviewers minimized the possibility of power-imbalance impacting students’ disclosures,^44^ and reflexivity practices were actively employed to acknowledge researcher positionality. The focus on the student perspective facilitated the identification of barriers and concerns regarding clinical empathy teaching in medical education as well as practical suggestions to address these while meeting students’ needs.

## Conclusion

Clinical empathy is a component of quality healthcare that influences patient outcomes and clinician wellness, making it an important priority in medical education. Ultimately, students perceived the effect of any clinical empathy-promoting educational intervention as highly contingent on its context and delivery. Students were able to offer insights into what curricular components they found beneficial and those that they believed were counterproductive to their expressed intent, highlighting the importance of including student voices in curricular innovation.

## Supplemental Material

Supplemental Material - Medical Student-Perceived Barriers, Facilitators, and Considerations Regarding Clinical Empathy Training: A Qualitative Study Supplemental Material for Medical Student-Perceived Barriers, Facilitators, and Considerations Regarding Clinical Empathy Training: A Qualitative Study by Shira Gertsman, MD, BSc, Anushka Nair, MD, BSc, Jason Wang, MD, BSc, Connie Li, MD, BSc, Sarah Klapman, MD, MSc, BMus, BA, Johanna Shapiro, PhD, Connie Williams, MD, PhD in Journal of Medical Education and Curricular Development.

Supplemental Material - Medical Student-Perceived Barriers, Facilitators, and Considerations Regarding Clinical Empathy Training: A Qualitative Study Supplemental Material for Medical Student-Perceived Barriers, Facilitators, and Considerations Regarding Clinical Empathy Training: A Qualitative Study by Shira Gertsman, MD, BSc, Anushka Nair, MD, BSc, Jason Wang, MD, BSc, Connie Li, MD, BSc, Sarah Klapman, MD, MSc, BMus, BA, Johanna Shapiro, PhD, Connie Williams, MD, PhD in Journal of Medical Education and Curricular Development.

## Data Availability

The participants of this study did not give written consent for their data to be shared publicly. Therefore, due to the sensitive nature of the research, supporting data is not available.
