# Supplementary material for: Medical Student-Perceived Barriers, Facilitators, and Considerations Regarding Clinical Empathy Training: A Qualitative Study
Source: J Med Educ Curric Dev. 2026 Sep 6;13:23821205261484413. doi: 10.1177/23821205261484413 (PMC13554687; doi:10.1177/23821205261484413)
Supplement: Supplementary file 2 [file sj-pdf-2-mde-10.1177_23821205261484413.pdf]

APPENDIX A: Research Team Experience, Positionality, and Reflexivity

The research team comprised five medical students and two supervising professors who had extensive experience with design, performance, and supervision of qualitative research. Focus group moderators completed a training course on focus group facilitation; SG, who was present at every group, has previously performed and published focus group research utilizing grounded theory methodology.

All members of the research team came to the project with lived experiences in medical education (as teachers and/or learners) that contributed to their interest in this project. The five medical student researchers practiced reflexivity by writing down individual memos and meeting frequently to discuss any challenges of focus group moderation and analysis. Memos and discussions involved critically reflecting on the influences of their own experiences as trainees on question-asking and data interpretation, and challenging biases related to positionality and preconceptions. The two supervisory team members were professors at medical schools, who confronted their own preconceptions as medical educators.

The researchers shared a constructivist paradigm which guided the philosophical assumptions underpinning the research, including that the researchers' positionalities were viewed as a crucial contributor to the construction of the study results. It was noted that our disciplinary orientations as medical learners/teachers would affect our approach to the research, understanding of the data, and interpretation of results. It was important for the learners to discuss experiences we were having actively in our training during data collection and analysis, which could prime us to interpret data in line with those experiences. The contributions of both teachers and learners in different roles within medical school provided diversity of perspectives regarding medical education.

The insider status of the focus group moderators as medical students appeared to prompt disclosure from participants, several of whom provided feedback that it felt like a safe space to discuss topics not frequently discussed in medical education. Although there are always power dynamics between researcher and participants, the "peer" dynamic helped avoid the power imbalance that exists between medical students and residents/attendings, while maintaining the mutual "understanding" of the topics at hand. However, it is possible that participants consciously or unconsciously were constrained by interviewers' statuses, especially in some cases where the interviewers and participants were from the same school (which participants were aware of in advance).

Team members also held individual assumptions about clinical empathy and its teachability. All members of the study team placed high value on clinical empathy, prompting their interest in this research topic. Considering this, participants were explicitly instructed during interviews to feel free to provide negative opinions/beliefs if applicable. During analysis, dissenting/contradictory opinions were highlighted. Student researchers discussed how their feelings about the distinction between "inherent" empathy and "taught" empathy became greyer as data collection and analysis progressed, reflecting on the

varieties of opinions and examples given by participants, and the integration of both concepts into the results.

The study supervisors were both educators of clinical empathy and, with their knowledge of the robust literature on the topic, believed that it is possible for clinical empathy to be taught and learned (note: supervisors were not directly involved in interviews or coding/data analysis). At the onset of project development, the entire research team met to discuss the nature of empathy, including inherent versus learned aspects, examining our individual ideas and incorporating those reflections into development of the study question and design. The overarching belief that empathy can be teachable in the right circumstances led us to investigated *how* empathy can be influenced by medical education rather than *if* is it. However, due to the nuances raised our discussions and debates, we included questions in the interview guide that asked participants whether they thought that empathy teaching truly translated to real life. This became particularly relevant in one focus group, in which a participant disagreed with the group and felt strongly that empathy is not teachable. This focus group was debriefed by the moderators, where we reflected on how our preconceptions could influence our openness to their perspective, and where we committed to adequately considering outlier thoughts in the analysis.

APPENDIX B: Semi-structured focus group guide

Semi-structured focus group guide

[Note: General prompting phrases such as "Would you like to elaborate on that?" will be used throughout at the moderator's discretion. The question list will act as a guide and not all questions may be asked in every group; questions may be modified, reordered, or reworded depending on participants' responses.]

The concept of empathy is defined differently by different people, but many consider it to be feeling another person's emotions. Clinical empathy, which is what we'll be focussing on today, is somewhat different than that, as it probably wouldn't be ideal to feel the emotions of every patient we see. Clinical empathy is defined as **a physician's ability to understand their patient's illness experience, communicate that understanding to the patient, and act on that understanding when providing care.** *[type this into Zoom chat in case participants wish to reference it, provide opportunity for questions]*

We would like to talk about your experience with your institution's current curriculum when it comes to teaching you aspects of clinical empathy. This includes formal teaching points that you may have learned in lectures, tutorials, or other dedicated classes that your school may have implemented. It also includes informal aspects of your training, such as observing preceptors during different clinical encounters. We encourage you to use personal experiences and examples wherever you feel comfortable and elaborate on the thoughts behind your answers as much as you like.

Focus Group Questions

**Theme**	**Questions**
Current curriculum ("What you are 'supposed' to be learning")	• Do elements of your curriculum explicitly address aspects of "clinical empathy"; that is, "the ability of the physician to understand the patient's whole illness experience, communicate this understanding, and subsequently act on this understanding to create a treatment plan with the patient"?<br>• How effective did you find this? Does it feel genuine?
	**Preclerkship**<br>• How do non-clinical aspects of your preclerkship curriculum (e.g. courses, group work, etc.) inform your motivation and ability to be a physician who tries to understand the patient's experience of illness?<br>• How have elements of your pre-clerkship curriculum addressed clinical empathy?
	**Clerkship**<br>• Consider times when you felt you were able to provide really good care to your patients. What was it about these interactions that defined and facilitated optimal care?<br>• What moments during clinical rotations have moulded your understanding of how to understand the patient's experience of illness?

Hidden curriculum (“What you are really learning”)	• Can you think of any times when you have seen physicians/residents acting with clinical empathy? Can you think of times when you have *not?*<br>o What do you think enabled and/or prevented the clinician from acting with clinical empathy?<br>• What do you feel your preceptor expects of you when you interact with patients? These may be unspoken expectations.<br>• Why do you think these are the expectations?<br>• Does the way the preceptor treats you impact how you treat patients?
Importance of clinical empathy	• Is it important that you act with clinical empathy *(repeat definition)*? If so, why?<br>o What do you think might act as an obstacle for you to act in this way?<br>o Is there something in the curriculum that you feel has improved your ability to act in this way?<br>• There is a lot of evidence that care is improved when physicians try to understand their patients. Do you see yourself doing this?<br>• What is your perception of how your school’s administration, academic leaders, and clinical teachers prioritize teaching and modeling empathy?<br>o Do you think it should be prioritized more or less?
Improving clinical empathy training	• There is evidence that clinical empathy decreases during training. Why do you think this is? Do you think this is avoidable (and if so, how)?<br>• Can you think of any ways to better instill empathy in medical learners?<br>o What do you feel your institution should add to their curriculum to help you connect better with patients?<br>• Do you find that what you are learning translates to real life?<br>o What aspects of real life encourage clinical empathy? What aspects of real life work against it?

[Provide an opportunity for any last questions/comments ABOUT clinical empathy. Then say after recording there will be a debrief.]

APPENDIX C: Demographics Questionnaire

The following form is anonymous, so we cannot tell who filled it out. By completing the form below, you are providing consent for us to use this information anonymously to report our overall study demographics.

1. Focus group number
2. Which medical school do you attend?
   - Michael G. DeGroote School of Medicine (McMaster University)
   - Northern Ontario School of Medicine
   - University of Ottawa
   - Queen's University
   - Temerty Faculty of Medicine (University of Toronto)
   - Schulich School of Medicine & Dentistry (Western University)
3. What stage of medical school are you currently at?
   - Preclerkship
   - Clerkship, early (not yet in final year of medical school)
   - Clerkship, late (in final year of medical school)
   - Other, please clarify: __________
4. Gender identity
   - Man
   - Woman
   - Non-binary
   - Two-spirit
   - Other, please specify (optional): __________
   - Prefer not to say
5. Age range
   - <20
   - 20-24
   - 25-29
   - 30-34
   - 35-39
   - 40+
   - Prefer not to say
6. Do you identify as a member of a visible minority/minorities or other marginalized group(s) (e.g. Indigenous, Black, POC, 2SLGBTQ+, person with a disability)
   - Yes
     - If you're comfortable, please describe how you identify: __________
   - No
   - Prefer not to say
7. How would you rate your overall satisfaction with your medical education so far?
   - Likert scale from 1-10, (1 = not satisfied at all, 10 = completely satisfied)

APPENDIX D: Summaries of code groups sent to participants for member-checking

Communication to Build Rapport

1. Establishing a good relationship with patients allows for clinical empathy to take place.
2. Keeping patients well-informed about their situation builds stronger relationships.
3. Miscommunication can lead to decreased empathy from the care provider and impairs the patient/physician relationship.
4. Listening and validating a patient sometimes provides more care than medical intervention.

Concerns Around Insincerity of Clinical Empathy

1. Active use of empathetic statements may in some cases come off as forced/insincere.
2. Just because a healthcare professional communicates with empathetic statements to the patient does not necessarily mean the provider is truly being empathetic or delivering care to reflect this.
3. Because empathy can be insincerely expressed, it is difficult to assess whether or not interventions meant to strengthen provider empathy are truly effective.

Desensitization Leading to Reduced Empathy

1. Repeated exposure to similar clinical encounters reduces clinical empathy when those cases are re-presented.
2. It is harder to build empathy in higher acuity situations (e.g. labour and delivery, emergency surgeries).

Difference Between Pre-Clerkship and Clerkship

1. The fast-paced nature of clerkship rotations makes it more difficult to learn and apply empathy compared to pre-clerkship.
2. Given the variable nature of different preceptors/rotations in clerkship, the quality of clinical empathy education varies greatly compared to pre-clerkship.
3. In general, pre-clerkship places a heavier emphasis on being empathetic than in clerkship.

Disconnect Between Teaching Empathy and Reality

1. It is awkward to be empathetic during OSCEs because it feels fake.
2. Empathy is emphasized in education but putting it to practice is limited.
3. Teaching empathy didactically is not useful because it needs to be demonstrated in clinical settings.

Empathy for Self-Benefit

1. Being empathetic towards patients may help practitioners maintain motivation throughout their medical careers.
2. The increased number of positive interactions that arise from a practitioner being more empathetic may help counteract burnout.

Empathy of Practitioner Affecting Patient Care

1. The degree of empathy a practitioner feels towards a patient may directly correlate to the overall quality of care they receive.
2. Being empathetic contributes less to overall patient outcomes in certain types of clinical encounters.
3. Regardless of the actual quality of care being provided, patients are generally more pleased with their care from a provider that displays empathy.
4. In some cases, just the act of having their stories heard can be independently beneficial for patients.
5. Possessing clinical empathy can help providers better anticipate the needs of their patients.
6. Sometimes, external factors impede our ability to be empathetic, which in turn directly harms the care patients receive.
7. Not every patient values the empathy of their practitioner the same way.

Importance of Debriefing Clinical Encounters

1. Preceptors who make space for debriefing simultaneously teach empathy and demonstrate their own.
2. Sometimes it is too difficult to debrief difficult clinical experiences when you have to continue working.
3. Support systems inside and outside of medicine are important for debriefing.
4. Designated debriefing sessions are important.

Importance of Testing Empathy Curriculum

1. Testing reinforces knowledge and testing on empathy teaching highlights its value in the curriculum.
2. Empathy is frequently tested through OSCEs.

Inherent Empathy of Care Provider

1. Empathy is a baseline personality trait that may be difficult to teach or increase.
2. People who go into medicine are often empathetic at baseline.
3. Medical students are often very empathetic and willing to express this with patients.
4. Truly empathetic practitioners incorporate empathy into their daily patient care, even when conditions are difficult.

5. Medical training can put a strain on students’ inherent empathy, leading to burnout and trauma.
6. Empathetic people like medical students need to be treated with empathy as well.

Lack of Incentive to Demonstrate Empathy

1. Empathy training can feel like an afterthought in the medical school curriculum.
2. Empathy for patients is emotionally draining and can lead to burnout.
3. Patients with challenging behaviours make acting with empathy more difficult.
4. Empathy takes time that it is not always possible to afford a patient, logistically and financially.
5. Some patients do not want empathetic doctors.

Lack of Supports Decreases Empathy

1. Medical students feel that, as they progress through their training, they feel less supported by administrators and there are fewer opportunities to connect as a class because everyone is scattered across sites; this contributes to burnout.
2. Having an external support system is necessary to prevent burnout and increase capacity for practicing clinical empathy.
3. Some residents are happier than others due to the culture of their program and the wellness opportunities and social outlets available to them.

Logistics of Teaching Clinical Empathy

1. Having consistent classes on empathy was effective.
2. Empathy can be taught in short encounters and with small gestures.
3. Making additional empathy training optional may help avoid overwhelming or alienating students, but also may result in low attendance by those who need the training most.
4. Having patients share their experiences is a good way to teach empathy.
5. Theoretical lessons on empathy are not as effective as teaching in clinical settings.
6. Empathy is not integrated well into the preclerkship curriculum.

Models of Clinical Empathy

1. The FIFE model supports clinical empathy and is highly emphasized.
2. Other models, like the SPIKES model and the Kleinman model, are also taught.

Patient Experiences in Building Empathy

1. Hearing real patients speak about their experiences is often the best way to grasp the nuances of what people with certain diseases go through, compared to didactic teaching or self-studying.
2. Seeing patients react positively/negatively to interactions with other providers builds upon students’ clinical empathy.

Practitioner Empathy Affects Rest of Healthcare Team

1. A healthcare provider's understanding/attitude towards a patient may significantly influence the empathy and care that other members of the healthcare team provide.
2. In particular, a provider in more of a leadership role may influence the attitudes of others, overall workplace culture, and the perceptions of learners working under them.
3. A provider's empathy towards patients may also influence how others in the workplace perceive the provider.

Role of Burnout in Empathy

1. It is hard to provide empathetic care in a strained healthcare system due to increased levels of burnout.
2. Physician burnout reduces capacity to practice clinical empathy.
3. It is hard to have empathy for patients when you are struggling yourself.
4. As burnout increases throughout years of training, empathy decreases.
5. Time constraints and desensitization to patient encounters increase burnout, which decreases empathy.
6. Having supports that decrease burnout allow for more capacity to build empathy.
7. Compassion fatigue from patient care leads to burnout.

Role of Hidden Curriculum

1. There is often pressure from preceptors/healthcare settings in general for learners to focus on efficiency and medical care instead of demonstrating clinical empathy.
2. The extra time with patients and lower clinical expectations that learners are often afforded early on in their education is beneficial for building clinical empathy.
3. The emotional wellbeing of patients is often valued less than their physical wellbeing, which also creates a hostile environment for providing empathetic care.
4. Many preceptors do not comment/pay attention to the lack of empathy in an interaction unless it directly leads to a hostile interaction of some kind with the patient.
5. Empathy is sometimes not taught/emphasized by preceptors because it is a barebones expectation of all learners to be empathetic.
6. Different schools/nations/cultures may have different expectations and views regarding empathy.

Role of Intersectionality in Empathy Training

1. Introducing intersectionality into empathy training is a key component of understanding the needs of patients from diverse backgrounds.
2. Different cultures may perceive/value clinical empathy differently from their healthcare providers.

3. There may be some dissonance between the way intersectionality is portrayed/taught in a structured medical school curriculum compared to the realities of clinical practice.
4. Education around intersectionality is not necessarily valued the same by all medical students, and even if the opportunities were available to learn more about the topic not everyone would be interested in participating.

Role of Medical Specialty in Empathy Training

1. Medical specialties have differing cultures involving differing levels or approaches to empathy.
2. It can be challenging for students to move amongst specialties with different expectations of empathy for patients.
3. Psychiatry and palliative care rotations tend to place more emphasis on patient empathy than more acute specialties like surgery or emergency.
4. The amount of time you are able to spend with patients directly correlates to the amount of empathy you can provide, and this varies by specialty.
5. Expectations for student interviews with patients, a site of possible empathy for patients, also vary by specialty.

Role of Observational Learning to Build on Own Perspectives

1. You are more likely to use skills you've learned through observation in practice as they are more memorable and impactful.
2. Observing the encounter through the patient perspective (patient panels, accompanying patients to appointments) is very effective in building empathy.

Role of Preceptors in Empathy Training

1. Clerkship learning experiences are more preceptor-dependent than pre-clerkship.
2. Demonstrating empathy depends on how your preceptors model empathy.
3. Sometimes preceptors "fake" empathy, which is challenging for students to navigate.
4. Truly empathetic preceptors listen to students to help provide better patient care.
5. Preceptors can make students miserable, which makes it hard to provide empathetic care.
6. Preceptors being empathetic to students helps make students empathetic to patients.
7. Preceptors who explain the patient experience to students help build empathy.
8. Preceptors who are honest about their limitations give more empathetic care.
9. It is more useful to see a preceptor model empathy than to be given theoretical empathy training.

Role of Simulation in Empathy Training

1. Using standardized patients is the best modality we have at this time to simulate patient interactions and practice using clinical empathy in training.
2. Although using standardized patients is helpful, it can be difficult to simulate real-life interactions because it does not feel as genuine.

Time Constraints in Expressing Empathy

1. The pressure to be efficient in collecting information during patient encounters does not allow for enough time to express empathy.
2. Due to the high cognitive load experienced by medical students, most of the time students have is spent on learning clinical information rather than building clinical empathy.
3. Higher patient-to-physician ratios decrease time that can be used to express empathy.
4. Spending more time with patients increases ability to practice clinical empathy.
5. It is hard to provide empathetic care in a strained healthcare system because of increased time constraints.
